# Supplementary figures and images for: A multicenter, randomized, open-label, controlled trial to evaluate the efficacy and tolerability of hydroxychloroquine and a retrospective study in adult patients with mild to moderate coronavirus disease 2019 (COVID-19)
Source: PLoS One. 2020 Dec 2;15(12):e0242763. doi: 10.1371/journal.pone.0242763 (PMC7710068; doi:10.1371/journal.pone.0242763)

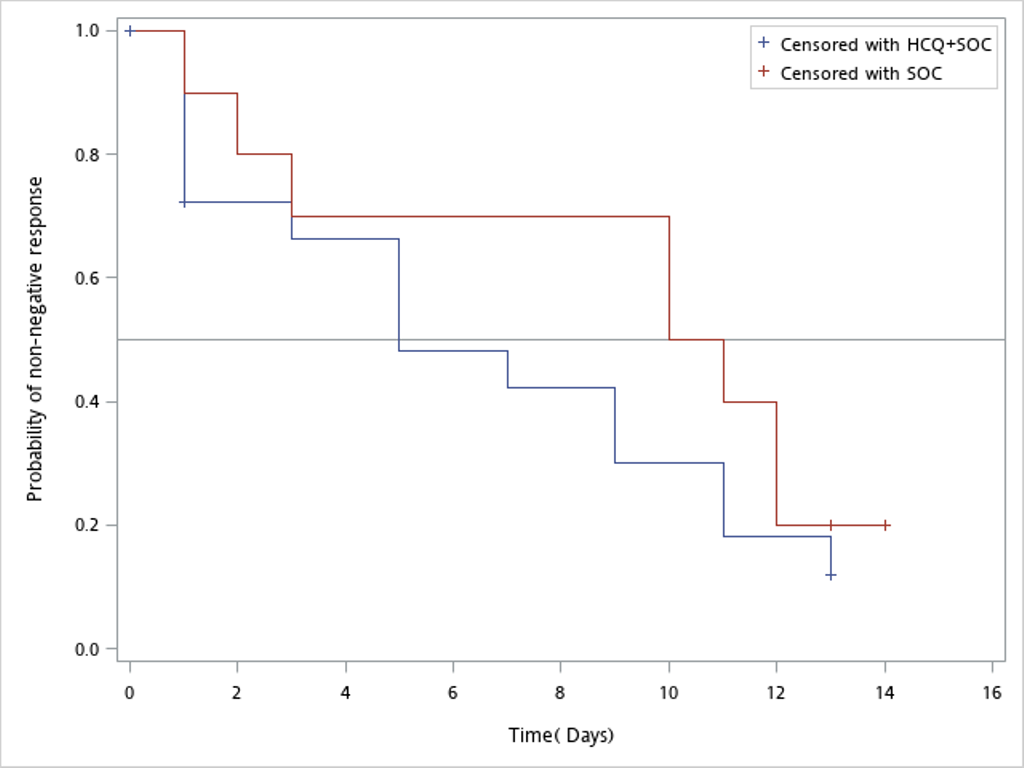

Supplement: S1 Fig — Abbreviations: HCQ: hydroxychloroquine; SOC: standard of care. (TIF) [file pone.0242763.s002.tif]

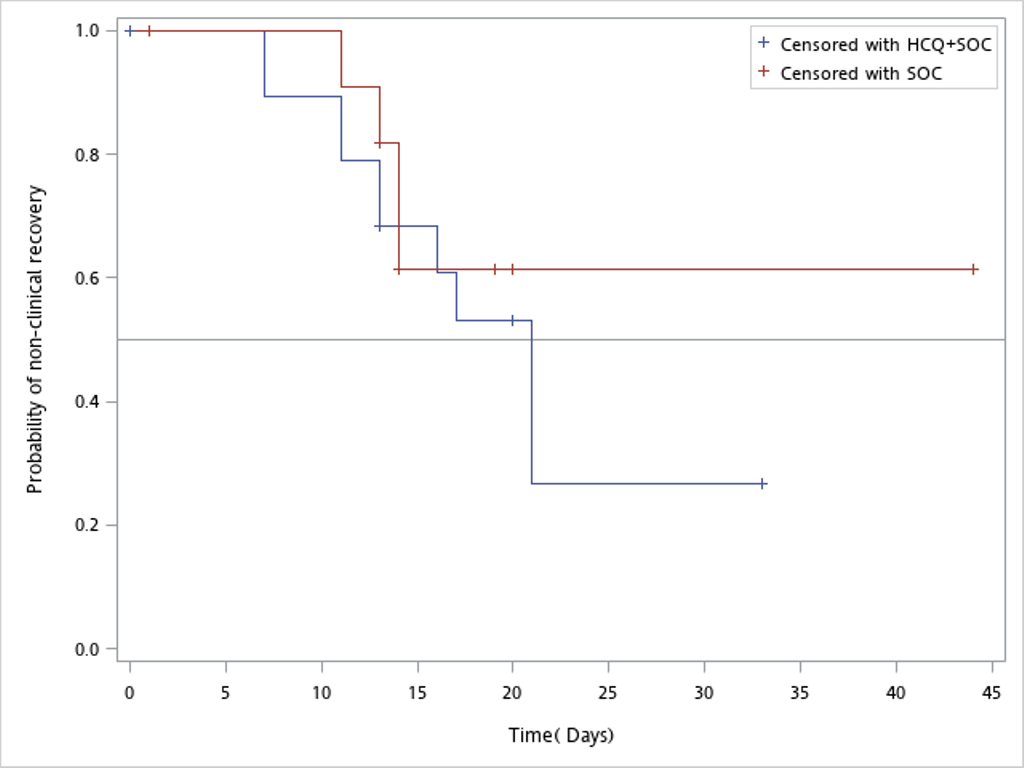

Supplement: S2 Fig — Abbreviations: HCQ: hydroxychloroquine; SOC: standard of care. (TIF) [file pone.0242763.s003.tif]
