# Supplementary material for: A multicenter, randomized, open-label, controlled trial to evaluate the efficacy and tolerability of hydroxychloroquine and a retrospective study in adult patients with mild to moderate coronavirus disease 2019 (COVID-19)
Source: PLoS One. 2020 Dec 2;15(12):e0242763. doi: 10.1371/journal.pone.0242763 (PMC7710068; doi:10.1371/journal.pone.0242763)
Supplement: S1 Table — (DOCX) [file pone.0242763.s004.docx]

**S1 Table. Comparison of median times and negative viral rRT-PCR results between subjects in the HCQ and SOC groups presenting with mild symptoms in the multicenter, open-label, randomized controlled trial.**

| Group | N | Negative^a^ | Median time to negative^b^  (Days, 95% CI)^c^ | *p*-value^d^ |
| --- | --- | --- | --- | --- |
| HCQ^e^ | 19 | 15 (78.9%) | 5 (1, 11) | 0.31 |
| SOC^f^ | 10 | 8 (80.0%) | 11 (1, 12) |  |

^a^Negative event: both pharyngeal swab and sputum showed negative results; ^b^Time to negative = Event date or censored date – start day; ^c^*p*-value: Log-rank test; ^d^CI: confidence interval; ^e^HCQ: hydroxychloroquine; ^f^SOC: standard of care.
