# Supplementary material for: A multicenter, randomized, open-label, controlled trial to evaluate the efficacy and tolerability of hydroxychloroquine and a retrospective study in adult patients with mild to moderate coronavirus disease 2019 (COVID-19)
Source: PLoS One. 2020 Dec 2;15(12):e0242763. doi: 10.1371/journal.pone.0242763 (PMC7710068; doi:10.1371/journal.pone.0242763)
Supplement: S2 Table — (DOCX) [file pone.0242763.s005.docx]

**S2 Table. Comparison of times to clinical recovery between subjects in the HCQ and SOC groups in the multicenter, open-label, randomized controlled trial**

| Group | N | Clinical recovery^a^ | Median time to clinical recovery^b^  (Days, 95% CI)^c^ | *p*-value^d^ | *p*-value^e^ |
| --- | --- | --- | --- | --- | --- |
| HCQ^f^ | 21 | 6 (28.6%) | .(9, .) | 0.54 | 0.51 |
| SOC^g^ | 12 | 5 (41.7%) | .(5, .) |  |  |

^a^Clinical recovery: 1^st^ time of 3 consecutive PCR tests showed negative and major symptoms showed negative; ^b^Time to clinical recovery = Event date or censored date – start day; ^c^CI: confidence interval; ^d^Log-rank test; ^e^Log-rank test stratified by clinical syndromes; ^f^ HCQ: hydroxychloroquine; ^g^SOC: standard of care.
