# Supplementary material for: A multicenter, randomized, open-label, controlled trial to evaluate the efficacy and tolerability of hydroxychloroquine and a retrospective study in adult patients with mild to moderate coronavirus disease 2019 (COVID-19)
Source: PLoS One. 2020 Dec 2;15(12):e0242763. doi: 10.1371/journal.pone.0242763 (PMC7710068; doi:10.1371/journal.pone.0242763)
Supplement: S3 Table — (DOCX) [file pone.0242763.s006.docx]

**S3 Table. Proportions of negative rRT-PCR assessments on day 14 and median times to negative rRT-PCR results in the multicenter, retrospective study**

| Group | N | Negative*_a_ | *p*-value^b^ | Median time to negative^c^  (Days, 95% CI)^d^ | *p*-value^e^ |
| --- | --- | --- | --- | --- | --- |
| HCQ^f^ | 28 | 12 (42.9%) | 0.70 | 15 (6, 31) | 0.37 |
| Control | 9 | 5 (55.6%) |  | 14 (7, 22) |  |

^a^Negative event: both pharyngeal swab and sputum showed negative results; ^b^Fisher’s exact test; ^c^Time to negative = Event date or censored date – start day; ^d^CI: confidence interval;

^e^Log-rank test stratified by clinical syndromes; ^f^HCQ: hydroxychloroquine.
