# Supplementary material for: A multicenter, randomized, open-label, controlled trial to evaluate the efficacy and tolerability of hydroxychloroquine and a retrospective study in adult patients with mild to moderate coronavirus disease 2019 (COVID-19)
Source: PLoS One. 2020 Dec 2;15(12):e0242763. doi: 10.1371/journal.pone.0242763 (PMC7710068; doi:10.1371/journal.pone.0242763)
Supplement: S1 File — (PDF) [file pone.0242763.s007.pdf]

Title Page:

Title: **A Multi-center, Randomized, Open-label, Controlled Trial to Evaluate the Efficacy and Tolerability of Hydroxychloroquine (HCQ) in Adult Patients with Mild to Moderate Coronavirus Disease (COVID-19) Compared to Standard of Care Treatment**

Principal Investigator:

Taoyuan General Hospital, Ministry of Health and Welfare: Shu-Hsing Cheng

Principal Investigators and Sub-principal Investigators at each hospital:

1 Taoyuan General Hospital, Ministry of Health and Welfare (311): Chien-Yu Cheng、Yi-Chun Lin, Cheng-Pin Chen

2 Keelung Hospital, Ministry of Health and Welfare (223): Chin-Feng Lin、 Jiing-Chyuan Luo、Fu-Shun Tsai、Tsung-Yen Yang、Wen-Chen Yau、Hon-Lai Wong

3 Taipei Hospital, Ministry of Health and Welfare (222): Wu-Pu Lin、Lin-Chen Chien、Chen-Han Yiu、Chien-Yu Huang、Yung-Tsung Hsiao

4 Miaoli General Hospital, Ministry of Health and Welfare (307): Ming-Huei Lee、Sz-Rung Huang

5 Taichung Hospital, Ministry of Health and Welfare (413): Wei-Sheng Chung、Tsung-Chia Chen、Ting-Yu Tseng

6 Feng Yuan Hospital, Ministry of Health and Welfare (414): Wei-Yao Wang、Yih-Farng Liou、Chen-Feng Chiu

7 Chang Hua Hospital, Ministry of Health and Welfare (408):Yi-Wen Huang、Yang-Hao Yu, Tse-Hung Lin , Tze-Yen Chang

8 Nantou Hospital, Ministry of Health and Welfare (415): Hung-Chang Hung、Tzung-Fan Chuang、Jia-Hong Liao、Li-Yueh Yeh、Shu-Ming Huang

9 ChiaYi Hospital, Ministry of Health and Welfare (610) :Yuan-Der Huang、Shih-Tien Chen、Chi-Min Shin、Chung-Shin Liao

10 Tainan Hospital, Ministry of Health and Welfare (612): Yuan-Pin Hung、Chih-I Lee、Chun-Wei Chiu

11 Pingtung Hospital, Ministry of Health and Welfare (801): Shah-Hwa Chou、Cheng-Yu Kuo、Tz-Lun Hung、Hsin-Hui Wang

Synopsis

**計畫書日期**	2020年3月31日
**修訂日期**	2020年4月20日
**試驗編號**	V0120
**試驗標題**	一項多中心、隨機分配、開放、對照試驗，針對患有輕、中度新冠狀病毒肺炎之成年病患，評估Hydroxychloroquine (HCQ) 相較於標準照護治療的療效與安全性
**試驗中心**	傳染病指定隔離醫院及應變醫院
**臨床階段**	不適用
**試驗目的**	**主要試驗目的**<br>• 評估 HCQ 對 COVID-19 病患病毒檢測陰性轉換之療效<br>**次要試驗目的**<br>• 探討 HCQ 對 COVID-19 病患其他病毒檢測指標之療效<br>• 分析 HCQ 對 COVID-19 病患臨床病徵之療效<br>• 評估 HCQ 對 COVID-19 病患的安全性與耐受性
**試驗指標**	**主要試驗指標：**<br>• 上呼吸道樣本中 rRT-PCR 檢測新冠肺炎病毒 (SARS-CoV-2) 為陰性所需時間 (病毒檢測僅需定性:第0、2、4、6、8、10、12、14天)<br>**次要試驗指標：**<br>• 第8天病毒清除率 (Virological Clearance Rate)<br>• 病毒載量的曲線下總面積 (AUC of viral load, 病毒檢測需定量及標準化:第0、2、4、6、8、10、12、14天)<br>• 病人達到臨床恢復 (Time to Clinical Recovery, TTCR) 所需時間<br>• 第14天病人出院比率<br>• 中樞神經症狀發生頻率<br>• 呼吸道症狀惡化頻率<br>• 病人於第二週與第三週的死亡率<br>• 嚴重不良事件頻率
**隨機分配病患總人數**	45位
**各治療組病患人數**	以1：2分配並利用輕、中症狀分層，15位標準照護、30位第一天口服 HCQ 400 mg每日兩次，第二至七日口服 HCQ 200

	mg 每日兩次，同時也接受標準照護。
**診斷**	患有輕、中度新冠肺炎之成年人
**主要納入條件與排除條件**	**納入條件：**<br>1. 年滿 20 歲至79歲以下的成年男性或女性<br>2. 病患有發燒 (≥38°C) 或急性呼吸道症狀，且經 rRT-PCR 檢測確定為新冠肺炎之成年人<br>3. 新冠肺炎為輕、中度<br>4. 願意且能夠遵從試驗程序並簽署書面受試者同意書<br><br>**排除條件：**<br>1. 已知對試驗藥物 HCQ 或其成份過敏或有過敏反應<br>2. 已知病人有嚴重肺臟、肝臟、腎臟、腦部、造血系統或其他重要的系統性疾病<br>3. 已知病人有視網膜、聽覺喪失或嚴重神經與精神性疾病<br>4. 已知病人有胰臟炎疾病<br>5. 篩選時從心電圖 (ECG) 看出控制不佳但具有臨床意義的心臟傳導異常病史、篩選時發現任何長 QT 波症候群或男性 QTcF 間隔 ≥> 450 毫秒、女性 ≥> 470 毫秒 (根據 Fridericia 的校正公式) 的病史或證據<br>6. 已知的人類免疫缺陷病毒感染、未同時接受現行治療的活性 B 型或 C 型肝炎（B 型肝炎檢測結果為陽性 [HBsAg 及 HBeAg 皆為陽性]，或高效價 C 型肝炎核糖核酸 [RNA] > 800,000 IU/ml）<br>7. 同時有控制不佳且不穩定的病情，包括依主持人判斷會影響患者安全、干擾試驗目標，或影響患者遵照試驗要求的蠶豆症精神疾病及酒精/藥物依賴/濫用<br>8. 使用改變HCQ 吸收或排泄併用藥物的患者<br>9. 患者被試驗主持者認定無法完成，或不適合此研究<br>10. 懷孕或哺乳女性
**試驗藥品**	Hydroxychloroquine (HCQ) 200 mg/Tab
**劑量**	第一天服用HCQ 400 mg 每日兩次，第二至七日 HCQ 200 mg 每日兩次
**給藥方式**	口服

Table of Contents

目錄

Synopsis 2
1. INTRODUCTION 5
2 ELIGIBILITY ASSESSMENT AND ENROLLMENT 5
3 STUDY IMPLEMENTATION 8
4 CONCOMITANT MEDICATIONS/MEASURES 11
5 BIOSPECIMEN COLLECTION 11
6 DATA COLLECTION AND EVALUATION 11
7 SAFETY REPORTING REQUIREMENTS/DATA AND SAFETY MONITORING PLAN 12
8 STATISTICAL SECTION 15
9 COLLABORATIVE AGREEMENTS 15
10 PHARMACEUTICAL AND INVESTIGATIONAL DEVICE INFORMATION 15
11 REFERENCES 16
12 APPENDIX (IF ANY) 17

1. INTRODUCTION

1.1 BACKGROUND AND RATIONALE:

On December 31, 2019, an outbreak of respiratory illness later proved to be caused by a novel coronavirus, officially named Coronavirus Disease 2019 (COVID-19), was notified first in Wuhan, a city of Hubei province, People's Republic of China (PRC). COVID-19 rapidly spreads in China and to other parts of the world. Currently more than 370,000 laboratory-confirmed cases have been reported worldwide, and the case count has been rising daily, and caused a global health emergency. As of March 29, 2020, there were 298 confirmed cases in Taiwan.

The effective medical treatment against COVID-19 infection is still unknown. Chloroquine phosphate is a well-known antimalarial drug which has been on the market for many years. Recently, *in vitro* study shown that Chloroquine is effective at both entry and at post-entry stages of the COVID-19 infection of Vero E6 cells with promising results. Chloroquine is also an immune-modifier and could distribute to the whole body including lung. Also, chloroquine is cheap and safe, and could be a promising agent against COVID-19 infection. However, only hydroxychloroquine (HCQ) with the extra hydroxyl group is available in Taiwan. Therefore, hydroxychloroquine instead become the best choice for the treatment candidate, since it shows higher *in vitro* potency (EC50) against COVID-19 with lower toxicity while retaining the original effect which compared with chloroquine.

1.2 STUDY OBJECTIVES

1.2.1 Primary Objective:

- To evaluate the efficacy of HCQ, with respect to the time to negatively RT-PCR assessments in COVID-19 patients.

1.2.2 Secondary Objective:

- To evaluate the efficacy of HCQ in the aspect of virologic assessments in COVID-19 patients (e.g. viral load and clearance)
- To explore the efficacy of HCQ regarding the change of clinical symptoms in COVID-19 patients (e.g. TTCR; proportion of discharge, CNS and respiratory symptoms; mortality)
- To evaluate the safety and tolerability of HCQ in COVID-19 patients

2 ELIGIBILITY ASSESSMENT AND ENROLLMENT

2.1 ELIGIBILITY CRITERIA

2.1.1 Inclusion Criteria

1. Male and female age between 20-79 years
2. Patients who had fever (central temperature ≥38°C) or acute upper respiratory symptoms and laboratory confirmation (rRT-PCR) for COVID-19, with available same type of upper respiratory tract specimens from screening evaluation to the initial testing within 4 days of initial testing
3. Patients have mild (no pneumonia) to moderate disease (pneumonia without respiratory distress) according to the following World Health Organization (WHO) definition of COVID-19 clinical syndromes:
   - <u>Mild (Mild illness):</u>
     Patients with uncomplicated upper respiratory tract viral infection, may have non-specific symptoms such as fever, fatigue, cough (with or without sputum production), anorexia, malaise, muscle pain, sore throat, dyspnea, nasal congestion, or headache. Rarely, patients may also present with diarrhoea, nausea and vomiting.
   - <u>Moderate (Pneumonia):</u>
     Adult with pneumonia but no signs of severe pneumonia and no need for supplemental oxygen.
4. Willing and able to comply with the study procedure and sign a written informed consent

2.1.2 Exclusion Criteria

1. Patients with the medical history of hypersensitivity to chloroquine, chloroquinine, or hydroxychloroquine
2. Patients with retinal disease, hearing loss, severe neurological and mental illness
3. Patients with pancreatitis
4. Patients with severe lung, liver (alanine aminotransferase (ALT)/aspartate aminotransferase (AST) elevation more than 3 times the normal upper limit), kidney (estimated glomerular filtration rate [eGFR] <30 mL/min/1.73m^2, using the MDRD or CKD-EPI methods), brain, haematological diseases or other important systemic diseases
5. Medical history of uncontrolled but clinically significant abnormal cardiac conduction abnormalities at electrocardiogram (ECG) at screening, any history or evidence of long QT syndrome or QTcF interval >450 msec for males and >470 msec for females (according to Fridericia's correction) at screening
6. Known HIV infection; active hepatitis B or C without concurrent treatment (positive tests for hepatitis B [both HBsAg and HBeAg], or high titer of hepatitis C ribonucleic acid [RNA]

>800,000 IU/ml)

7. Uncontrolled and unstable concurrent medical condition including G6PD, psychiatric disorders and alcohol/substance dependence/abuse that will jeopardize the safety of the patient, interfere with the objectives of the study, or affect the patient compliance with study requirements, as determined by the Investigator
8. Patients with concomitant use of medications that alter the absorption or excretion of hydroxychloroquine
9. Patients were considered to be unable to complete the study, or not suitable for the study judged by Investigators
10. Pregnant or breast-feeding women

2.2 SCREENING EVALUATION

Patient who had fever (central temperature ≥ 38°C) or acute upper respiratory symptoms and are tested positive for COVID-19 from respiratory specimen by rRT-PCR methods. According to “the Interim Guideline for the Clinical Management of New Coronavirus (SARS-CoV-2) Infection”, the throat swabs, sputum (lower respiratory tract fluid) and serum should be collected while the initial test for confirmation of infection suspected candidate. The same type of respiratory specimens should be collected and assessed for eligibility confirmation in screening evaluation of the study. In addition, Hemogram, biochemistry, urinalysis, chest X ray and ECG will be checked. Above screening and baseline evaluation could be done before signing the written informed consent form as clinically needed.

2.3 RANDOMIZATION (OR STRATIFICATION) PROCEDURES (APPLICABLE FOR RANDOMIZED CONTROLLED TRIALS)

After signing the written informed consent form, 3-digits screening number contains the first letter of S (starting from S01) will be assigned to subject sequentially. Subject screened but not eligible will have the screening number only.

Subjects who meet all study criteria after the screening evaluation will be randomly assigned to in a 2:1 ratio, stratified with mild illness (no pneumonia) and moderate disease (with pneumonia), to receive hydroxychloroquine or have standard of care (SOC) treatment. A randomization number (starting from 001) will be sequentially assigned to an eligible subject. Replacement subjects will be assigned the corresponding number, starting from 101 (i.e., the replacement subject for 005 will be assigned a number of 105, etc.).

2.4 Baseline Evaluation

Baseline assessment of participants will be evaluated as follows (data collected at screening could be considered baseline): Chest X ray, throat swabs and sputum collection for COVID-19. Hemogram, biochemistry, urinalysis, and ECG, and been considered as baseline. Every effort will be made to collect all types of specimen (throat swabs, sputum, lower respiratory tract fluid, or serum) for rRT-PCR assessment from screening patients before day 1. For evaluation of primary efficacy endpoint, the type of respiratory tract specimens in each assessment day should be the same with baseline.

3 STUDY IMPLEMENTATION

3.1 Study Design

This is a multi-center, randomized, open-label, controlled trial to evaluate the efficacy and tolerability of Hydroxychloroquine Sulfate (HCQ) in adult Patients with mild to moderate coronavirus disease (COVID-19) compared to standard of care treatment (SOC). The primary endpoint for the study is to evaluate the efficacy of HCQ, with respect to the time to negatively rRT-PCR assessments from the randomization date up to 14 days. The secondary endpoint is to evaluate the efficacy of HCQ in the aspect of virological assessments and the change of clinical symptoms. In addition, the safety and tolerability of HCQ will be evaluated during treatment period in COVID-19 patients.

The study will employ an open-label parallel design, to evaluate the efficacy and safety between hydroxychloroquine sulfate (plaquenil) and controlled standard of care treatment. The administration plan of HCQ is 400 mg bid on Day 1 and 200 mg bid for 6 days on Day 2-7. All of the enrolled subjects will receive standard of care. The comparison group will receive standard of care, i.e., supportive treatment for subjects with mild COVID-19 clinical illness, and antimicrobial therapy for subjects who developed moderate COVID-19 clinical illness (ceftriaxone 2gm daily for 7 days ± azithromycin 500mg on Day1 and 250mg on Day2-5; or levofloxacin 750 mg daily for 5 days, or levofloxacin 500mg daily or moxifloxacin 400mg daily for 7-14 days for the subjects who had allergic history to ceftriaxone or azithromycin, or per physician's discernation). Oseltamivir 75 mg bid for 5 days will be given for subjects who have concomitant influenza A or B infection. The subjects will be randomly assigned to a 2:1 ratio to receive HCQ + SOC, or SOC alone. The estimated case number will be 30:15 and will be a competitive enrolment at 11 sites. No dose reduction, modification, or change in the frequency of HCQ will be recommended during the study period.

The investigators may determine to switch to HCQ treatment arm on Day 8 if the subjects

developed progression of pneumonia clinically, or roentegenographically, or had persistent high viral load without 10 log of decline, compared to baseline data.

An independent data monitoring committee (IDMC) will be instituted to ensure external objective medical and/or statistical review of efficacy to protect the ethical interests and well-being of participants and to protect the scientific validity of the study.

3.2 Drug Administration

The investigational product will be administered by direct observation.

3.3 Dose Modifications

No dose reduction, modification, or change in the frequency of hydroxychloroquine will be recommended during the study period.

3.4 Study Calendar

Schedule of Visits

Procedure	***Screening /Baseline***		***Schedule day***									
		Day 1	***Day 2***	***Day 4***	***Day 6***	***Day 7***	***Day 8****	***Day 10****	***Day 12****	***Day 14****	***Follow – up (day 21)***	
ICF	X											
History and Eligibility	X	X										
Randomization	X											
Vital signs	X	X	X	X	X	X	X	X	X	X		
Physical examination	X	X	X	X	X	X	X	X	X	X		
Hematology/Biochemistry(CBC+DC,PT, aPTT, CRP, ESR, GOT/GPT/TBIL,Cr, eGFR, Na, K, Cl, Ferritin, d-Dimer, CPK, LDH, hs-Troponin I)	X			X			X		X			
Serology test (stored for antibody tests)	X			X			X		X			
Urinalysis	X											
rRT-PCR assessment of respiratory tract specimen (throat swabs, sputum. Stool if diarrhea)	X		X	X	X		X	X	X	X		
CNS symptoms assessment	X	X	X	X	X	X	X	X	X	X		
Assessment of SpO_2,, or PaO_2/FiO_2 if any	X	X	X	X	X	X	X	X	X	X		
Radiological Assessments (X-ray)	X			X			X		X			
EKG	X			X			X					
Pregnancy test	X											
HBsAg, HBeAg and HCV Ab tests	X											
Survival check										X	X	
HCQ/SOC treatment		X	→	→	→	→						
Adverse Events	X	X	X	X	X	X	X	X	X	X		
Concomitant Medications	X	X	X	X	X	X	X	X	X	X		

Footnote* Study completed if 3 consecutive negative rRT-PCR results

3.5 Criteria for Removal from Protocol Therapy and Off Study Criteria

3.5.1 Off-Study Criteria

Participants who developed adverse events including grade 3/4 liver toxicity, grade 3 allergic reaction, or other severe adverse events (including progression to the stage of severe pneumonia)

4 CONCOMITANT MEDICATIONS/MEASURES

Concomitant medications will be recorded well.

5 BIOSPECIMEN COLLECTION

5.1 Sample Storage, Tracking and Disposition

All of the biospecimens are considered biohazard, and should be managed according to Taiwan CDC's guidelines. Store serum will be shipped to designated laboratory, and will be destroyed if no more extended studies permitted at the end of 2020 (appendix 4).

6 DATA COLLECTION AND EVALUATION

6.1 Data Collection

The study will use eCRF/EDC for data collection. Safety laboratory data will be entered into eCRF by authorized personnel at Investigational sites. Real-time RT-PCR data will be managed and stored within the laboratory information management system and only the date and time of specimens are recorded in the eCRF. Safety laboratory data will be integrated with the consolidated clinical data before database lock.

All records should be kept in conformance to applicable national laws and regulations.

Adverse events will be recorded according to the Division of AIDS (DAIDS) Table for Grading the Severity of Adult and Pediatric Adverse Events(Appendix 3), and will be coded using the current MedDRA thesaurus (Version 19.0); concomitant medication will be coded using World Health Organization Collaborating Centre for Drug Statistics Methodology Anatomical Therapeutic Chemical/defined daily dose (WHOCC ATC/DDD).

6.2 Criteria of primary endpoints

6.2.1 Primary efficacy endpoints

- Transition time of SARS-CoV-2 nucleic acid rRT-PCR from positive results to negative results in respiratory tract specimen on day 0, 2, 4, 6, 8, 10, 12, 14. For evaluation of primary efficacy endpoint, the type of respiratory tract specimens in each assessment day should be the same with Day 0.

6.3 Secondary Endpoints

6.3.1 Evaluation of Efficacy

- Virological clearance rate at day 8
- AUC of viral load on day 0, 2, 4, 6, 8, 10, 12, 14

  For the above 2 secondary efficacy evaluations, the type of respiratory tract specimens in each assessment day should be the same with Day 0.

- Time to Clinical recovery (TTCR):
  TTCR is defined as the time (in hours) from randomization (active or placebo) until normalization of fever, respiratory rate, and oxygen saturation, and alleviation of cough, sustained for at least 72 hours.

  <u>Normalization and alleviation criteria:</u>
  - Fever - ≤36.9°C or -axilla, ≤37.2 °C oral,
  - Respiratory rate - ≤20/minute on room air,
  - Oxygen saturation - ≥ 94% on room air,
  - Cough - mild or absent on a patient reported scale of severe, moderate, mild, absent

- Frequency of CNS symptoms (e.g. headache, dizziness, impaired consciousness, ataxia, acute cerebrovascular disease, and epilepsy)
- Frequency of respiratory progression:
  Defined as SpO_2 < 94% on room air or PaO_2/FiO_2 < 300 mmHg and requirement for supplemental oxygen or more advanced ventilator support
- Proportion of Participants Discharged by Day 14
- The mortality rate of subjects at week 2 and week 3

6.3.2 Evaluation of Safety

- Frequency of serious adverse events

6.4 Toxicity Criteria

7 SAFETY REPORTING REQUIREMENTS/DATA AND SAFETY MONITORING PLAN

7.1 Definitions

7.1.1 Adverse Event

An adverse event is defined as any reaction, side effect, or untoward event that occurs during the course of the clinical trial associated with the use of a drug in humans, whether or not the event is considered related to the treatment or clinically significant.

7.1.2 Suspected adverse Event

Suspected adverse reaction means any adverse event for which there is a <u>reasonable</u> <u>possibility</u>

that the drug caused the adverse event. For the purposes of IND safety reporting, 'reasonable possibility' means there is evidence to suggest a causal relationship between the drug and the adverse event. A suspected adverse reaction implies a lesser degree of certainty about causality than adverse reaction, which means any adverse event caused by a drug.

7.1.3 Unexpected adverse reaction

An adverse event or suspected adverse reaction is considered "unexpected" if it is not listed in the investigator brochure or is not listed at the specificity or severity that has been observed; or, if an investigator brochure is not required or available, is not consistent with the risk information described in the general investigational plan or elsewhere in the current application. "Unexpected", also refers to adverse events or suspected adverse reactions that are mentioned in the investigator brochure as occurring with a class of drugs or as anticipated from the pharmacological properties of the drug, but are not specifically mentioned as occurring with the particular drug under investigation.

7.1.4 Serious

An adverse event or suspected adverse reaction is considered serious if in the view of the investigator or the sponsor, it results in any of the following:

- Death,
- A life-threatening adverse drug experience
- Inpatient hospitalization or prolongation of existing hospitalization
- Persistent or significant incapacity or substantial disruption of the ability to conduct normal life functions
- A congenital anomaly/birth defect.
- Important medical events that may not result in death, be life-threatening, or require hospitalization may be considered a serious adverse drug experience when, based upon appropriate medical judgment, they may jeopardize the patient or subject and may require medical or surgical intervention to prevent one of the outcomes listed in this definition.

7.1.5 Disability

A substantial disruption of a person's ability to conduct normal life functions.

7.1.6 Life-threatening adverse drug experience

Any adverse event or suspected adverse reaction that places the patient or subject, in the view of the investigator or sponsor, at immediate risk of death from the reaction as it occurred, i.e., it does not include a reaction that had it occurred in a more severe form, might have caused death.

7.1.7 Unanticipated Problem

Any incident, experience, or outcome that:

- Is unexpected in terms of nature, severity, or frequency in relation to

  (a) the research risks that are described in the IRB-approved research protocol and informed consent document; Investigator's Brochure or other study documents, and

  (b) the characteristics of the subject population being studied; **AND**
- Is related or possibly related to participation in the research; **AND**

- Places subjects or others at a g*reater risk of harm* (including physical, psychological, economic, or social harm) than was previously known or recognized.

7.2 REC OR IRB REPORTING

Unexpected AE, SAE will be reported. And interim analysis will be reported to IRB when 22 participants are enrolled.

7.3 DATA AND SAFETY MONITORING PLAN

DSMB will monitor the safety of the study, and the committee will be held every 2-4 weeks aftr 1st participant enrolled.

7.3.1 Principal Investigator/Research Team

All protocols should include a DSM plan which describes how the investigator plans to oversee research subject safety and ensure data integrity. The PI and his/her research staff are part of the monitoring plan but may not be the only ones conducting monitoring activities for any given protocol.

The clinical research team will meet on a regular basis {*insert frequency*} when patients are being actively treated on the trial to discuss each patient. Decisions about dose level enrollment and dose escalation if applicable will be made based on the toxicity data from prior patients.

All data will be collected in a timely manner and reviewed by the principal investigator or a lead associate investigator. Adverse events will be reported as required above. Any safety concerns, new information that might affect either the ethical and or scientific conduct of the

trial, or protocol deviations and violations will be immediately reported to the IRB using iRIS and if applicable to the Sponsor.

The principal investigator will review adverse event and response data on each patient to ensure safety and data accuracy. The principal investigator will personally conduct or supervise the investigation and provide appropriate delegation of responsibilities to other members of the research staff.

7.3.2 Data Safety Monitoring Board (DSMB)

A DSMB is an impartial group established to oversee a clinical trial and review the results to determine if they are acceptable. Members of a DSMB must be multidisciplinary and include members with relevant clinical and statistical expertise. The DSMB should meet at least annually or more often depending on the activity and nature of the clinical trial being monitored.

This protocol requires monitoring by the Data Safety Monitoring Board (DSMB) as described above in Section 7.3. Interim outcome results will not be revealed to the investigators of the trial; results will be presented to the investigators prior to final accrual to the trial only if the DSMB recommends early termination of the trial. (NOTE: the study statistician is responsible for providing the description of how the monitoring will take place, including endpoints to be monitored and the frequency or timing of monitoring.)

8 STATISTICAL SECTION

Data will be listed and summarized using SAS® V 9.2 or higher (SAS Institute, Inc., Cary, North Carolina) according to Sponsor-agreed reporting standards, where applicable. Complete details will be documented in the statistical analysis plan (SAP).
All efficacy and safety data will be summarized using descriptive statistics by treatment. The following descriptive statistics will be used to summarize the trial data on the basis of their nature unless otherwise specified:

- Continuous variables: number of non-missing observations, mean, standard deviation, median, minimum, and maximum.
- Categorical variables: frequencies and percentages.
- Time-to-event variables: number of non-missing observations (N), median, minimum, and maximum. Kaplan-Meier event rates may also be provided if applicable for specific time to event variables.

9 COLLABORATIVE AGREEMENTS

9.1 MULTI-INSTITUTIONAL GUIDELINES

9.1.1 IRB Approvals

9.1.2 Amendments and Consents

10 PHARMACEUTICAL AND INVESTIGATIONAL DEVICE INFORMATION

10.1.1 Source: Taiwan Biotec Co., LTD.信東藥廠

10.1.2 Formulation and preparation: See Appendix 1

10.1.3 Toxicity: See appendix 2

10.1.4 Stability and Storage: room temperature and avoiding heat and moist

10.1.5 Administration procedures: oral

10.1.6 Incompatibilities: See Appendix 2

11 REFERENCES

1.World Health Organization. Novel coronavirus situation report -65. Geneva, Switzerland: World Health Organization; 2020. https://www.who.int/docs/default-source/coronaviruse/situation-reports/20200325-sitrep-26-ncov.pdf?sfvrsnZfb6d49b1_2. [Accessed 25 MAR 2020].
2. Cheng SC, Chang YC, Fan Chiang YL, Chien YC, Cheng M, Yang CH, Huang CH, Hsu YN. First case of Coronavirus Disease 2019 (COVID-19) pneumonia in Taiwan. J Formos Med Assoc. 2020 Feb 26. pii: S0929-6646(20)30044-9. doi: 10.1016/j.jfma.2020.02.007. [Epub ahead of print]
3. Huang C, Wang Y, Li X, Ren L, Zhao J, Hu Y, Zhang L, Fan G, Xu J, Gu X, Cheng Z, Yu T, Xia J, Wei Y, Wu W, Xie X, Yin W, Li H, Liu M, Xiao Y, Gao H, Guo L, Xie J, Wang G, Jiang R, Gao Z, Jin Q, Wang J14, Cao B.Clinical features of patients infected with 2019 novel coronavirus in Wuhan, China. Lancet. 2020 Feb 15;395(10223):497-506.
4. Yasri S, Wiwanitkit V. Editorial: Wuhan coronavirus outbreak and imported case. Adv Trop Med Pub Health Int 2019;9:1-2.
5. Zhang W, Du RH, Li B, Zheng XS, Yang XL, et al. Molecular and serological investigation of 2019-nCoV infected patients: implication of multiple shedding routes. Emerg Microbes Infect. 2020 Dec;9(1):386-389. doi: 10.1080/22221751.2020.1729071.
6. Cortegiani A, Ingoglia G, Ippolito M, Giarratano A, Einav S. A systematic review on the efficacy and safety of chloroquine for the treatment of COVID-19.J Crit Care. 2020 Mar 10. pii: S0883-9441(20)30390-7. doi: 10.1016/j.jcrc.2020.03.005. [Epub ahead of print]
7. Gautret P, Lagier JC, Parola P, Hoang VT, Meddeb L, et al. Hydroxychloroquine and azithromycin as a treatment of COVID-19: results of an open-label non-randomized clinical trial. Int J Antimicrob Agents. 2020 Mar 20:105949. doi: 10.1016/j.ijantimicag.2020.105949. [Epub ahead of print]

12 APPENDIX

12.1 Drug photography

12.2 Drug information

12.3 DAIDS tables for adverse event grading for severity

12.4 Stored serum preparation
